# Designing Multifunctional Urban Green Spaces: An Inclusive Public Health Framework

**DOI:** 10.3390/ijerph191710867

**Published:** 2022-08-31

**Authors:** Andrew J. Lafrenz

**Affiliations:** School of Nursing & Health Innovations, University of Portland, 5000 N. Willamette Blvd, Portland, OR 97203, USA; lafrenz@up.edu

**Keywords:** urban green space, nature and health, forest therapy, urban design, multifunctional green space

## Abstract

Evidence of the wide range of health benefits associated with the use of urban green space (UGS) continues to grow. Despite this evidence, many UGS designs do not adopt a community-inclusive approach that utilizes evidence-based public health strategies to maximize potential health benefits. This research focused on testing a multidisciplinary, community-involved public health framework to drive the UGS design process. The aim of this study was to use community feedback and evidence-based public health practices to promote physical health, psychological wellbeing, and social cohesion by creating a multifunctional UGS that enhances nature therapy, natural play, and sports and recreation. Community health assessment data (236 survey responses), community forum and survey feedback (157 survey responses), local urban green space inventory assessment, and environmental assessment and impact data were analyzed to develop a design plan that maximize the greatest potential health benefits for the greatest proportion of the population. Community health data indicated a strong relationship between the availability of places to be physically active in the community and higher ratings of mental (aOR = 1.80) and physical (aOR = 1.49) health. The creation and utilization of the proposed community-inclusive and public health-focused framework resulted in a UGS design that prioritized the needs of the community and provided evidence-informed strategies to improve the health of local residents. This paper provides unique insight into the application of a framework that promotes a more health-focused and functional approach to UGS design.

## 1. Introduction

As the population density increases in many cities around the world, urban green spaces (UGS) become increasingly important as areas to promote a wide range of health benefits. The World Health Organization (WHO) has stated that urban green spaces are a “necessary component for delivering healthy, sustainable, livable conditions” and have urged urban planning to include more evidence-based public health approaches [1]. The scientific research overwhelmingly supports the substantial and growing evidence of the influence green spaces has on multiple aspects of physical and psychological wellbeing. As proposed by Veen et al. [2], the majority of health benefits that UGS help promote can generally be grouped into three distinct categories of health benefits: (1) physical health, (2) psychological wellbeing, and (3) social cohesion [2]. The benefits to physical health are supported by studies that show an association between greater exposure to green spaces and parks and higher levels of physical activity in children and adults [3,4], lower levels of obesity in children and adults [5,6], improved sleep quality in adults [7], decreased cardiovascular disease incidence [8,9], and decreased Type 2 diabetes incidence [10]. The benefits to psychological wellbeing are supported by studies that show higher levels of green space exposure to be associated with improved mental wellbeing, overall health, cognitive development in children [11], lower psychological distress in teens [12], and lower risk of a wide spectrum of psychiatric disorders later in life for those with higher levels of continuous green space presence during childhood [13]. In addition, studies have indicated that time spent in forests is associated with lower cortisol levels [14] and a reduction in reported feelings of hostility, depression, and anxiety among adults with acute and chronic stress [15].

Despite our understanding of the importance of green spaces to human health, green space is under increasing pressure from growing urban populations and the associated urbanization processes of expansion and densification [16]. With the available green and blue spaces decreasing in many communities, the importance of maximizing natural areas and parks for their health and wellness benefits should be a priority. When looking at the pathways in which UGS affect health, the benefits have been attributed to: (1) being physically active in nature or (2) being present in nature. However, the characteristics of the space itself is also influenced by its functionality [17]. How and with whom (e.g., alone or with others) individuals use the UGS also influence its potential benefits [2].

**Figure 1:**
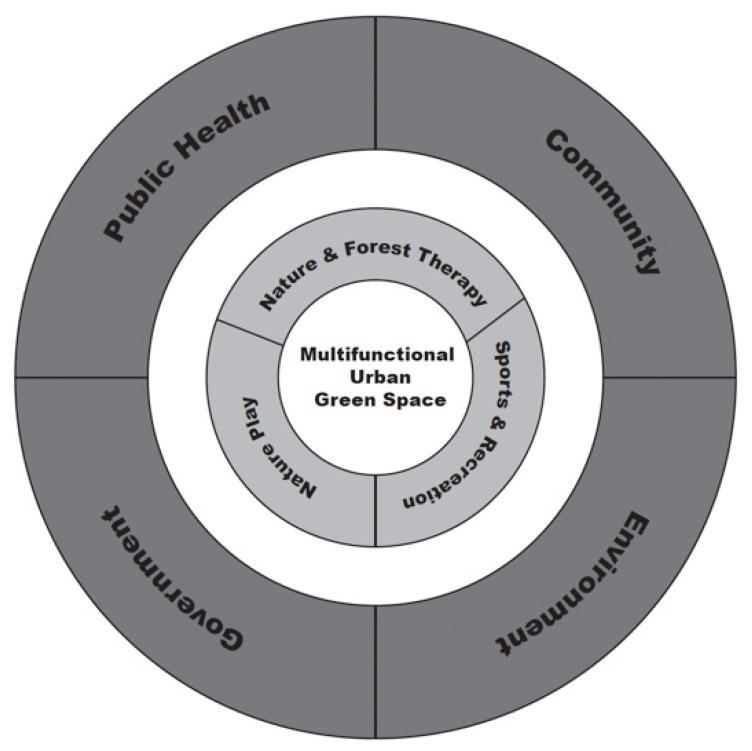
Proposed multidiscipline, multifunctional urban green space design framework.

This paper will present a case study on the utilization of a multidisciplinary urban green space design framework (Figure 1) that includes four components: (1) local government officials, (2) local community members, (3) local public health professionals, and (4) local environmental experts. While researchers continue to focus on the health benefits of being active and present in nature, little research has focused on how public health scientists and a diverse representation of local community members can work collaboratively with urban planners to develop healthier, more livable, and more environmentally sustainable communities. The research question central to this study was: can a community-inclusive, public-health-focused design framework improve the multifunctionality and therefore the potential health benefits of an urban green space?

### 1.1. The Problem

Despite the growing evidence of the relationship between green spaces and a variety of health benefits, urban planners rarely design multifunctional spaces that can provide all three distinct health benefits related to improving: (1) physical health, (2) psychological wellbeing, and (3) social cohesion in the same space. The historical model in the U.S. has been for local governments to make smaller green spaces available in traditional neighborhood parks with sports fields, bike paths, playground structures, and picnic tables. Alternatively, larger green spaces are often set aside as natural areas with little to no infrastructure and a focus on allowing individuals to be in nature. As such, these different types of green spaces typically target specific populations depending on their amenities. For example, parks primarily designed with playground structures are mostly visited by families with young children. Parks with sports and athletic fields are mostly visited by older children and adolescents. Lastly, nature areas are primarily visited by older adults. Less common, is the design of green spaces that function as: (1) natural play structures for young children, (2) sports and athletic fields for school-aged children, and (3) areas that provide nature and forest-therapy features for all ages. With a body of research now shedding light on the multiple pathways by which green spaces and parks can affect our health [18,19], urban planners have yet to adopt strategies that consider these multiple pathways. Creating multifunctional green spaces would maximize the potential health benefits for the greatest number of individuals in a community. Ensuring that UGS design maximizes the numerous health, social, and environmental benefits is critical. In order to accomplish this, urban planners should strive to be more inclusive and invite a greater number of community stakeholders and public health professionals to the table when designing green space functionality.

### 1.2. Aim of the Study

This paper aims to present: (1) a multidisciplinary, community-inclusive green-space-design framework, and (2) the results of incorporating a public health approach that informs the design of a green space by maximizing health benefits through multifunctionality.

### 1.3. Significance of the Study

This paper presents a case study of a multidisciplinary urban green space design approach and framework that is informed by public health research. To date, much of the research in the nature and wellness field has focused on providing evidence of the various health effects in different populations, understanding the health benefit pathways, or retrospective evaluation of the UGS built environment. Few studies have presented frameworks for how to create collaborative and effective UGS design teams that can maximize the evidence-based health benefits of green spaces.

## 2. Materials and Methods

This case study took place from December 2021 to July 2022. Data were obtained through community health assessment questionnaires, several community-distributed surveys, publicly accessible planning documents, and interviews with multiple organizations. Details of the data collection methods are given below. The framework developed was used to guide a new design approach for an urban green space in the city of Scappoose, in the state of Oregon in the United States. Scappoose is a small town in Oregon, the United States of America, with approximately 8010 residents. Traditionally settled as a farming, logging and fishing town, most residents now commute to work approximately 25 miles away in Portland, the largest city in the state of Oregon. The town of Scappoose, Oregon provides a unique case study on multifunctional urban green space design for several reasons: (1) Its proximity to a large metropolitan area (Portland, OR, USA) and its combination of urban and rural areas results in elements of an urban layout, but with more available public green space than many urban areas. (2) The green space involved in this study includes a significant number of valuable natural green and blue areas, which allows for a unique design for use as both a park and open natural area. While Scappoose is designated as both urban and rural (depending on the defining organization and the reason for designation) for the purpose of this study, the green space will be referred to as an urban green space (UGS) due to its location within the city limits and within a well-developed area of the small city.

**Figure 2:**
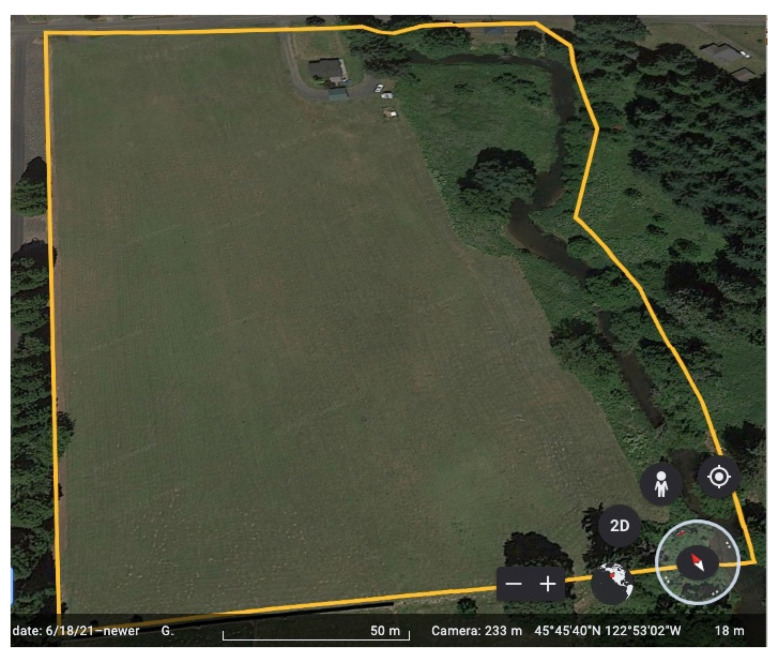
The undeveloped UGS boundaries and layout. (Google Earth 7.3, (2022) Scappoose Public Green Space, 45°45′36″ N 122°52′55″ W, elevation 13 M. [Online] Available at: http://www.google.com/earth/index.html [accessed on 30 July 2022]).

The specific UGS included in this case study is 9.54 acres in size (see Figure 2) and features a small stream that runs along the eastern border of the area. The stream, known as the South Scappoose Creek (SSC), is an important tributary of the Scappoose Bay Watershed, which drains into the Columbia River that borders Oregon and Washington in the United States. The SSC includes several endangered species of fish and other forms of wildlife, including coho and chinook salmon, and steelhead and cutthroat trout. Extensive efforts have been made to restore the many creeks and waterways within the Scappoose Bay Watershed due to the importance of the salmon habitat and water quality, and in order to mitigate the increasingly frequent local flooding events.

Overall, there were four teams that brought their own unique expertise to the UGS design framework: (1) local government officials focused on local land use policy and long-term green space, parks, and trails planning, (2) a public health scientist (the author) focused on the community health impact assessment, identifying measurable health outcomes, and presenting strategies to maximize the multifunctional properties of the green space in order to have the greatest health impact in the community, (3) a community parks and recreation committee that focused on gathering community feedback on what local residents wanted the green space to include, and (4) a local environmental group that focused on stream habitat restoration and flood plain improvements in the green space.

### 2.1. Demographics

Demographic data were obtained from the 2021 U.S. Census Report [20]. The median age in Scappoose is 41.3 years of age and, compared to both the United States and the surrounding county, Scappoose has a higher proportion of children (aged 14 years and under) and working-age adults (aged 25 to 44 years). The population includes 26% under 18 years of age and 18% over the age of 65 years, and 37% of all households have children under the age of 18 living with them, which is higher than both the surrounding county (34%) and the State of Oregon (30%). The average household size is 2.56 persons—also larger than the surrounding county (2.55) and Oregon State (2.47). Race and ethnicity demographics are: 87% white (non-Hispanic), 1% Asian, 2% American Indian, and 8% Hispanic.

### 2.2. Framework Variables

#### 2.2.1. Public Health Level

Data on health-related variables for local residents were obtained from a secondary dataset that was part of a larger tri-county community health assessment, completed by multiple counties and a health system, in the spring of 2022. The survey was modeled after the Center for Disease Control and Prevention’s Behavioral Risk Factor Surveillance System (BRFSS) which assesses health status, health risk behaviors, and healthcare access and utilization. The BRFSS is a well-established survey with strong validity and reliability. Electronic links to the surveys were posted on social media and included in newsletters, with a total of 236 responses collected and analyzed for this study. Health-related variables analyzed in this study included: the prevalence of chronic disease, perceived physical and mental health, depression and anxiety, social isolation, and perceived community physical activity options. Obesity rates were obtained from publicly available Behavioral Risk Factor Surveillance Systems (BRFSS) survey data for the year 2018 (CDC, 2022).

#### 2.2.2. Community Level

Community data were collected over the course of 18 months in several different formats. Three surveys were distributed electronically on social media, in newsletters, and in-person over this period to collect feedback on local parks and green spaces. Included were questions about how likely respondents were to use different amenities, such as athletic fields, playgrounds, dog parks, and nature trails, in this specific green space. In addition, several community forums were held where local residents could provide feedback to local officials about how they would like to see the green space designed. Lastly, local residents were also encouraged to attend monthly city council and park and recreation committee meetings to provide feedback on the design of the green space.

#### 2.2.3. Environmental Level

As part of the pre-planning for this green space, an extensive environmental assessment was completed by the local watershed council and external environmental consultants. From an environmental standpoint, this green space contained several critical environmental components that needed to be considered. The presence of a stream running the entire length of one side of the space required extensive flood-plain mitigation planning and endangered fish species habitat planning, as well as wetlands identification and preservation.

#### 2.2.4. Government Level

Local government design input included providing data that were focused on how the green space contributed to the long-term planning and development of the city. Data on the current park and green space inventory in the city were collected from the publicly available 2017 Scappoose Parks, Trails, and Open Spaces Plan. The report included valuable data on the current inventory of parks and green spaces, as well as undeveloped public land, for future park and green-space development. The local government also provided information on how the city master plan and future infrastructure improvements might affect the UGS design.

#### 2.2.5. Statistical Analysis

IBM SPSS^®^ for Windows^®^ version 24 (IBM Corp., Armonk, NY, USA) was used for data analysis. Bivariate relationships were explored using Pearson correlations. Logistic regression analysis was performed, and models produced to determine community indicators as predictors for high vs. low mental health and high vs. low physical health. Independent variables with a *p* < 0.05 in the bivariate analysis were included in the logistic regression model testing.

## 3. Results

### 3.1. Public-Health-Level Data

Demographic data are reported in Table 1 and general community health indicators are summarized in Table 2. Of note are the relatively high number of residents that reported two or more health conditions (60%), as well as 39% of residents reporting having anxiety or depression or both. Overall, 72% of Scappoose residents rated their mental health as good, very good or excellent (compared to 71% in the surrounding communities) and 76% rated their physical health as good, very good or excellent (compared to 80% in the surrounding communities). The prevalence of obesity among adults aged 18 years and older was 33% in the Scappoose community, with the same levels found in the surrounding communities. A relatively high percentage of the population reported that there were options for community physical activity (77).

Significant moderate correlations (r = 0.41–0.46) were seen between indicators of community livability, such as “my community is a good place to raise children” or “grow old”, and it “feels safe” and there are “places to be active nearby” (Table 3). Other Pearson correlation tests indicated significant low to moderate (r = 0.22–0.43) correlations between various physical and mental health indicators and having places to be physically active nearby (Table 4).

Final logistic regression models indicated a significant association between perceived places to be physically active in the community and physical health (aOR = 1.49) and mental health (aOR = 1.80), as shown in Table 5. No other independent variables were found to be significantly associated with physical and mental health, and therefore were not included in the final model as predictors. The final model was adjusted for age, gender, and race.

### 3.2. Community Level Data

Community feedback related to features that should be prioritized in the development of the new green space included 157 survey responses from community residents, and is summarized in Table 6. Overall, the survey responses strongly indicated that the availability of more nature trails and open spaces was a priority of the community. In addition, there was a strong response from the local soccer and softball community advocating for sports fields that could accommodate both sports. The community also ranked their top two recreational priorities as (1) walking and biking for exercise and (2) enjoying the outdoors and nature.

### 3.3. Environmental-Level Data

The local watershed council submitted a full stream restoration proposal to the city in May of 2022. A full description of the environmental component is not included, as the details of the plan fall outside the scope of this study. However, a summary of the environmental assessment and design plan will be discussed briefly due to its importance in developing the public health-focused design of the remaining green space. The environmental proposal was primarily used to help provide details on design constraints relating to the stream bank lay-back, and where a transition to the more traditional park amenities, such as play structures, athletic fields, and picnic tables, could occur. State and federal environmental regulations protect a large riparian buffer zone near the stream. However, a balance was achieved by designing a nature-therapy-focused trail, as well as several areas that provide access to and interaction with the green and blue areas around the creek.

### 3.4. Government- and Urban-Planning-Level Data

Data from the most recently completed parks, open spaces and trails report indicate that Scappoose currently has 2.93 acres of parkland for every 1000 residents. In comparison, the National Recreation and Park Association has established their benchmark for the level of service for a community to be 6.25–10.5 acres of parkland for every 1000 residents. The addition of this UGS will increase that ratio to 3.75 acres of parkland for every 1000 residents, moving Scappoose closer to the established national guidelines. The development of this UGS will also increase the number of residents that are within a walking distance of five miles to a park by an estimated 220 residents. Lastly, there is currently no park or green space within the Scappoose city limits that is designated as a natural open area. Additionally, the park and green space inventory indicated a disproportionately lower number of structures designed for under-2 year olds and the 2–5 year old age group. Sensory-friendly playground structures that are more accessible for children with autism and other challenges were also notably not present in this community.

## 4. Discussion

### 4.1. The Multifunctional Green Space Design Plan

The extensive work completed by the four components of the framework resulted in a comprehensive multifunctional UGS design proposal, which was submitted to the city. The purpose of this paper was to use this case study to provide insight into the application of this framework, including the strengths and challenges of a multidisciplinary team working on a community-involved, public-health-informed UGS design approach for improving health in the community. The scope of this article is not to provide details of the full UGS design plan, due to the variability and local context of each unique green space. However, an outline of the design elements will be discussed in the context of involving community members, public health professionals, and local city planners, as well as environmental experts. The main components of the proposal included an environmental habitat and stream restoration plan, athletic and sports fields and facilities, natural play zones, and a nature-therapy-focused path along the stream. These four main components of the UGS will maximize the potential health benefits through multifunctionality. The design was intended to provide health benefits by targeting opportunities to be active in nature, experience and interact with nature, and engage in social interactions in a park and natural area.

**Figure 3:**
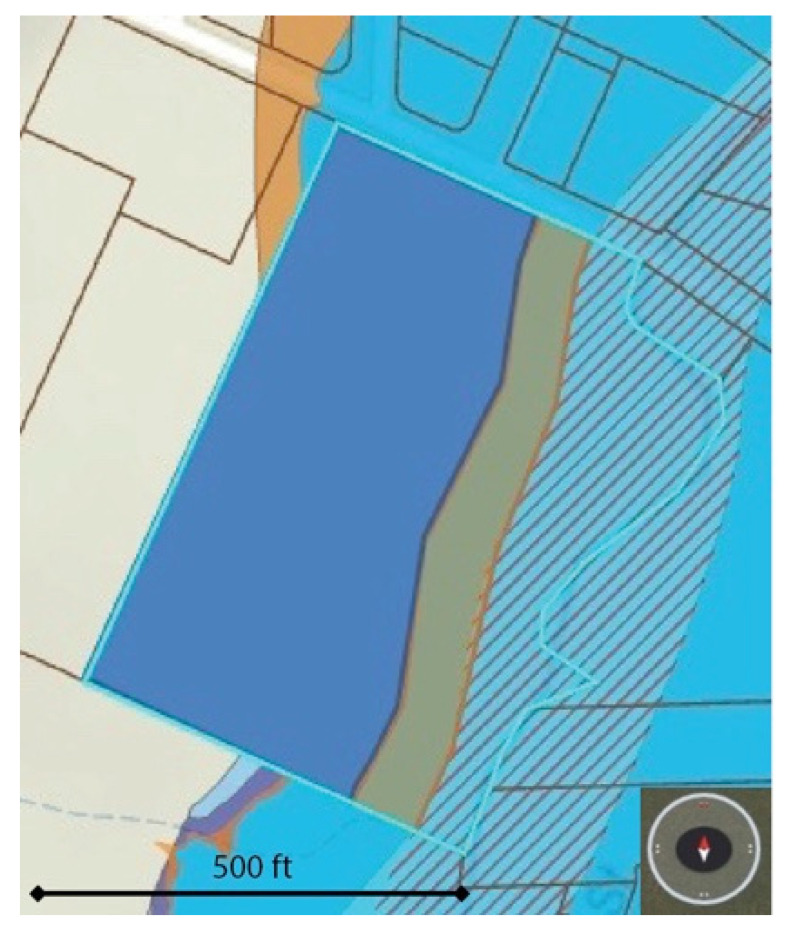
The three UGS zones are shown in this figure. Diagonal stripes represent the protected riparian buffer zone around the waterway. Orange represents the nature therapy and natural play transition zone. Blue represents the sports-and-recreation-dedicated area (Columbia County, OR, USA, GIS Mapping).

The completion of the environmental assessment and stream restoration plan was essential for understanding how much of the 9.54 acres would remain after restoring the natural flood plain. The original creek bank will be laid back to the required FEMA-designated regulatory floodway, as shown in Figure 3. As a result, approximately 2.2 acres along the creek will be set aside as a protected natural area. It is essential that future UGS design not only include improvements to the natural areas for habitat restoration and biodiversity, but also should include provisions for future climate-change-related health impacts. For example, in this green space design, significant benching of the creek bank will be completed in order to alleviate the increasingly more frequent flood events and associated risks to homes, buildings, and other infrastructure. In addition, mature trees will be preserved, which will help to provide shade and urban cooling as many areas around the world experience more frequent and more severe heat waves. A zone of approximately 1.5 acres along the border of the regulatory floodway will provide a transitional zone, designed for residents to interact with nature. In addition, there will be two water-access locations for children and adults to have access to the stream. This transitional zone is an important element in the multifunctionality of the green-space design. Rather than a hard delineation between protected natural areas and athletic fields and concrete bike paths, a softer and more inviting wood chip path is proposed; this path meanders around natural features such as trees, shrubs, and boulders. In addition, natural play structures are proposed, which combine the necessary water drainage requirements (bioswales) with additional natural play features for children to enjoy. Recent research indicates the additive health benefits of natural play for children, compared to traditional playground use. Brussoni et al. [21,22] found a significant decrease in depression and aggression post-nature play exposure/intervention, and another study found a positive increase in mood post-nature play exposure/intervention [23]. Other studies on natural play have shown improvements in cognitive development [24,25], learning [23,26], and social outcomes related to nature play [21,23]. Additional features in the transitional zone will include features intended to facilitate nature and forest therapy, such as natural boulder and log seating areas. The creation of “nature rooms”, made up of small spaces surrounded by mature trees and plantings with a high biodiversity and a variety of textures and colors, will be an important feature that invites individuals to pause and open their senses to all that nature has to offer. These design elements not only facilitate the formal sequences involved with nature and forest therapy but also are supported by research that demonstrates the health benefits associated with higher biodiversity in green spaces [27]. A dedicated zone that focuses on optimally facilitating nature and forest therapy is a unique feature of this UGS design. These features draw from the growing evidence of the benefits of guided nature and forest therapy. The growing practice of forest bathing, or nature and forest therapy, as it is more commonly known in North America, has highlighted the benefits of guided experiences in nature. A systematic review by Wen et al. [28] on the medical empirical research into forest bathing (*shinrin-yoku*) indicated that there was growing evidence of a wide range of health benefits, through both physiological effects and psychological effects [28].

After accounting for the stream and flood plain mitigation and the nature-wellness-and-therapy-focused paths and structures, there remained approximately 6 acres of space that could be designed for other health-related priorities. Based on the feedback from the community, the community health assessment, and the assessment of the current parks and green spaces, the remaining space focused on sports and recreation. Design recommendations included building a multiuse artificial-turf soccer and softball field. Compared to natural grass, artificial turf would better withstand the high levels of annual precipitation seen in this geographical location. Natural grass athletic fields are often unusable for large periods of the year in this location; therefore, an artificial turf surface would provide year round multifunctional use, leading to greater health benefits for a larger proportion of the community. Additionally, included in the public-health-informed design plan was an interactive play structure for children aged four years and under. Information from the park and green space inventory indicated that the city lacked dedicated play structures or environments for this age group, severely limiting the health benefits that outdoor play can have during this developmentally critical period in life. It was also recommended that the structures tailored towards younger age groups include covered areas to provide a longer window of use throughout the year and to be more inclusive of the needs of breastfeeding mothers. Adaptive play structures and environments was also recommended in order to be more inclusive of individuals with physical and sensory challenges. Lastly, covered picnic structures were recommended based on the evidence supporting the importance of community social cohesion that parks are able to provide [29].

### 4.2. The Importance of a Public Health Approach

The significant associations seen in Table 3 and Table 4, and the final adjusted regression model in Table 5, all highlight the relationship between places to be physically active and mental and physical health. This is supported by findings from other studies that show significant relationships between mental health outcomes [30] and proximity to green spaces, as well as physical health outcomes and green-space density [31]. Conducting a community health assessment at a local level is particularly beneficial for providing data to local government and stakeholders. Local health data provides valuable insight into the unique needs and priorities of each community, greatly improving the evidence supporting specific design elements of a particular UGS. Research have shown that public-health-focused approaches to green space interventions are more likely to improve health behaviors [32,33].

The timeline in which the four teams contributed to the UGS design framework also provided unique insights into the application of the framework. The public-health-informed design recommendations occurred after the government, community, and environmental teams submitted their design proposals. This allowed for an informal assessment of what the design plan would look like without the public health-informed guidance. It should be noted that this was not an intentional design of the methodology of the research study. Rather, it was a reflection of the local government’s general exclusion of public health guidance in the initial design of this UGS. As a result, this paper is able to provide a natural experimental perspective of how a UGS would have been designed with government oversight and community and environmental input, but without public health guidance. Prior to the public-health-focused design recommendations, the green space was proposed to be a general use park with athletic fields and an open grass field that stopped at the hard border of the stream riparian buffer zone. This design would have resulted in a green space that functioned strictly as a sports and recreation park and therefore did not meet the definition of multifunctionality. The inclusion of the nature-therapy transition zone is an important element that creates multifunctionality and targets additional groups for health benefits related to improving psychological wellbeing. In addition, the natural play structures and environments for the 0–4 age group was not present before the public health assessment of groups that lacked adequate opportunities for nature play in the city. Lastly, the public health framework identified a lack of any sensory and adaptive play equipment. Improving the accessibility of parks and green spaces was a top priority of the public health-focused design proposal.

### 4.3. Strengths of This Study

The significance of this paper to the literature on nature and public health is in the application of a multidisciplinary, community-involved, public health-informed framework for UGS design. The framework, as outlined in this study, include four areas of influence: (1) community involvement, (2) an evidence-based public health approach, (3) invested environmental groups, and (4) local government land use and planning officials. These four areas of influence were able to work collaboratively to design an UGS that can support the three main functions of (1) sports and recreation, (2) nature-based wellness for all ages, and (3) environmental improvements and sustainability.

The inclusion of the local community throughout the collaborative cocreation process was essential to ensure that the UGS was adapted to their needs, and that the prioritized health and wellbeing outcomes are achieved. Public health approaches and recommendations are also strengthened when developed in collaboration with what the community describes as its priorities [34]. Ultimately, partnerships between public health teams and community groups, such as in this study, are essential for maximizing the inclusivity, access, and utilization of green spaces.

Currently, much of the design and development of green spaces occur at the discretion of local government, with little or no community involvement or public health influence. Including local public health experts can serve several functions. (1) The design of different elements of parks and green spaces can be supported by evidence that they influence health-related behaviors and outcomes. (2) Local public health practitioners and scientists can assist with methods of conducting a health impact assessment to identify priority targets and the most effective use of the space for the greatest health impact. (3) Lastly, they can help to identify appropriate measurable outcomes and develop strong evaluation plans. Local public health departments are valuable resources for healthy urban planning partnerships, as they are particularly well versed in the current health needs and priorities of the communities in which they serve and live.

### 4.4. Challenges of This Study

While this study provides a template for an effective multidisciplinary design framework, there were several challenges. Firstly, an increase in the number of contributing teams added a level of complexity to traditional government-led green space design. Other challenges included organizing effective communication plans between the various contributing design groups. Working on roles, responsibilities, and communication strategies for design teams early on will ensure that a more cohesive planning process occurs. Lastly, it should be noted that there remains uncertainty as to how the work of all of these groups will be included in the final development of the UGS. Ultimately, it is the decision of the local city council and planning commission to finalize the design of the green space. While local city officials have responded favorably to the design components submitted by each design group, it has not yet been decided which elements will be included in the final UGS development. Urban planners must balance the range of competing demands, including housing demand, economic development, and long-term city planning, and recognize that optimizing green space for maximum health benefits is not always a priority [35].

### 4.5. Limitations and Future Research

While this paper provides an important case study of a community-involved, public-health-informed design approach for green spaces, there remain several limitations. The relatively small sample sizes of the community survey responses, as well as the health impact assessment, may result in health data and community park input that are not reflective of the greater community.

The moderately large size of the UGS in this case study allowed for enough physical space for a focus on all three priorities: (1) sports and recreational fields and spaces, (2) undeveloped, natural open spaces for nature play and nature therapy, and (3) wildlife habitat and stream restoration. Communities and local governments may face the challenge of working with much smaller green spaces when trying to maximize them and design for multifunctional use. However, this framework is not necessarily dependent on large green spaces, and can be applied to the design of relatively small green spaces. Research has shown that many of the health benefits related to being in nature can be achieved in relatively small natural environments. For example, South et al. [36] demonstrated in a cluster randomized trial that the “greening” of vacant lots reduced self-reported feelings of depression and worthlessness in the intervention group compared to the control group.

Future research should focus on approaches to multifunctional green space design that can be scaled down for smaller spaces such as “pocket parks”, which are smaller green spaces located throughout neighborhoods. Additionally, larger sampling of community health data, and community feedback on parks and green spaces, would ensure that larger communities are represented. The incorporation of theoretical models driving multidisciplinary UGS design would also improve our understanding of the relationship between UGS design-based interventions and their use and related health impacts.

## 5. Conclusions

This study provides important insight into how to develop community-involved, public health-informed design principles for a multifunctional green space. While every green space has unique contextual variables around its design and development, this paper provides a case study of how the needs of many groups in a community can be met while also restoring natural areas and stream and wildlife habitats. Furthermore, by including public health experts and the local community, the restoration of natural areas can in fact be inclusive of the important health benefits associated with human interactions in natural areas and spaces. The study also highlights the importance of community- and public health-involved frameworks in the design phases of green space development. While land use and development policies are primarily driven by local governments throughout much of the world, work needs to be undertaken to connect local government decision makers with public health scientists and community groups. With the amount of available urban green space declining in most communities, it is critical that efforts are made to make these areas as accessible as possible for a wide range of populations. Optimizing green spaces for sports and recreation as well as interactions with nature will ensure that communities can experience the many interrelated yet distinct health benefits of green spaces.

## Figures and Tables

**Table 1:** Demographic characteristics of respondents (*n* = 236).

Characteristics	*n*	Percent of Sample
Age		
18–40 years old	71	30%
41–64 years old	130	55%
65 years old and over	35	15%
Gender		
Female	146	62%
Male	73	31%
Non-binary/other	17	7%
Race		
White	191	81%
Multiracial	17	7%
American Indian	3	1%
Alaskan Native		
Asian	3	1%
Black/African American	2	1%
Other or Unknown	24	10%
Household makeup		
HH w/children < 18 yo	116	49%
HH w/adults > 65 yo	88	37%

**Table 2:** Community Health Indicators (*n* = 236).

Self-Reported Health Indicators	*n*	Percent of Sample
Good physical health *	179	76%
Good mental health *	170	72%
Anxiety or depression	91	39%
Cardiovascular risk factors	92	39%
One or more health issues	175	74%
Two or more health issues	142	60%

* Responded as “good”, “very good” or “excellent”.

**Table 3:** Correlations between community livability indicators and places to be physically active in the community.

	There Are Places in My Community to Be Physically Active
My community is a good place to raise children	0.46 *
My community is a good place to grow old	0.41 *
My community feels safe	0.42 *

* *p* < 0.01.

**Table 4:** Correlations between health indicators and places to be physically active in the community.

	There Are Places in My Community to Be Physically Active
Physical health rating	0.22 *
Mental health rating	0.32 *
Feeling loved and wanted	0.43 *
Feeling socially isolated	0.31 *
Feeling down, depressed, hopeless	0.29 *

* *p* < 0.01.

**Table 5:** Relationship between independent predictor “there are places to be physically active in my community” and mental and physical health ratings.

Health Outcome	Crude OR (95% CI)	Adj OR (95% CI)
Mental Health Rating		
High	1.74 (1.28–2.37)	1.80 (1.26–2.56)
Low	1	1
Physical health rating		
High	1.51 (1.11–2.02)	1.49 (1.06–2.08)
Low	1	1

**Table 6:** Community Green Space Survey (*n* = 157).

Question	*n*	Percent of Responses
The development of parks is important to me	146	93%
I would support more trails in Scappoose	127	81%
Scappoose parks do not meet my needs	113	72%
Parks are important when choosing where to live	133	85%

## Data Availability

The data presented in this study are available on request from the author.
